# Vegetable but not animal protein intake is associated to a better physical performance: a study on a general population sample of adults

**DOI:** 10.29219/fnr.v63.3422

**Published:** 2019-09-19

**Authors:** Diana Gazzani, Francesco Zamboni, Francesco Spelta, Pietro Ferrari, Veronica Mattioli, Lucia Cazzoletti, Elisabetta Zanolin, Stefano Tardivo, Marcello Ferrari

**Affiliations:** 1 Department of Diagnostics and Public Health, Unit of Hygiene and Preventive, Environmental and Occupational Medicine, University of Verona, Verona, Italy; 2 Department of Medicine, Unit of Respiratory Disease and Sports Medicine, University of Verona, Italy; 3 Department of Diagnostics and Public Health, Unit of Epidemiology and Statistical Medicine, University of Verona, Italy

**Keywords:** six minute walking test, nutrition, diet, exercise, proteins

## Abstract

**Background:**

The research was conducted in the frame of a population-based, case control study, called Genes Environment Interaction in Respiratory Disease.

**Objective:**

To assess the association between protein intake and physical performance in a general population sample.

**Design:**

Researchers investigated the association between the participants’ dietary information and their physical performance using the 6-min walking test and the distance walked in metres (6MWD) as main outcome measure. Information on dietary intake was collected using the validated European Investigation into Cancer and Nutrition food frequency questionnaires (FFQs). Then, daily intake of energy and macronutrients was estimated by means of the NAF software (nutritional analysis of FFQ). Linear regression models were used to evaluate the associations between vegetable, animal and total protein intakes and the 6MWD. The models were adjusted for socio-demographic features, total fats and available carbohydrate intakes.

**Results:**

The participants were 223 subjects (57% females) aged between 23 and 68 years. Their mean vegetable and animal proteins intake for gram/kg of body weight/day were, respectively, 0.4 and 0.7. After adjusting for all the potential confounders, there was a significant increase of 20.0 (95% CI 0.8; 39.2) m in the distance walked for an increase in 10 g/day of vegetable proteins and non-significant variations of −1.8 (95% CI −9.3; 5.7) m for an increase in 10 g/day of animal proteins and of 0.5 (95% CI −6.8; 7.7) for an increase in 10 g/day of total proteins.

**Discussion and conclusions:**

Our result suggests a positive role of vegetable proteins on physical performance. Whether this result is related to the high protein intake itself or may be a consequence of the other properties of plant-based foods deserves further investigation.

## Popular scientific summary

- Few studies have been conducted on the separate effect of animal and vegetable protein on physical performance.
- Analysing this relationship, we found an unexpected result, suggesting that a higher vegetable protein intake is associated with a better performance at 6-min walking test.
- Whether this result is related to the high protein intake itself or may be a consequence of the other properties of plant-based foods deserves further investigation.

A poor physical performance tested by objective measures of physical capacity seems to play a significant role in predicting an increased risk of mortality and morbidity in the general elderly adult population (1–3). In the last decades, the accumulation of evidence has provided new findings on the impact of the nutritional status on the health and functional capacity in the general population (4, 5). It is well known that an adequate intake of quality protein is a key factor for building, preserving muscle mass and maintaining physical functions. Currently, the recommended protein intake is 0.8 g/kg body weight/day (bw/day) for adults (6), even though this amount is a rough estimate, based on the minimal protein intake necessary to maintain the nitrogen balance in adults. It has recently been proposed that the protein intake of 1.0–1.2 g/kg bw/day is likely to be the amount that is required to ameliorate muscle health without damaging the renal function (4, 7). As regards the quality of proteins, it is a common opinion to favour those of animal origin since a greater proportion of daily protein intake derived from animal- versus plant-based sources seems to be associated with better muscle maintenance in older adults (8). Furthermore, animal proteins are more easily available and have a higher level of essential amino acids, which increases protein synthesis and anabolism (9, 10). Several previous investigations (11–15) have been conducted on the association between dietary protein intake and physical performance. Data from these studies generally support the effect of animal protein on preserving muscle mass and improving muscle strength in older adults (9, 16, 17). On the contrary, few studies have examined the association of a dietary protein intake, in terms of both quantity and quality, with physical performance measures in middle-aged adults (i.e. aged between 40 and 65 years). In view of the scarcity of evidence on this topic, the present study aimed at investigating the possible relationship between total animal and vegetable protein intake and the distance walked in 6 min in a cohort of subjects from the general population.

## Methods

### Study design

The Genes Environment Interaction in Respiratory Diseases (GEIRD) project is a multi-case-control study on respiratory diseases, carried out between 2007 and 2010 in Italy. The sample was randomly selected from the general population in six centres (Pavia, Sassari, Turin, Ancona, Terni and Verona) by using the local health authority records. The GEIRD project’s design is described in detail elsewhere (18). In brief, cases of chronic bronchitis, chronic obstructive pulmonary disease, asthma or rhinitis and controls without respiratory symptoms were identified through a two-step design (postal screening, clinical interview). During the clinical interview, subjects performed the 6MWT and filled in a food frequency questionnaire (FFQ). In the present analysis, only subjects without respiratory symptoms or diseases who participated in the study in Verona, with valid information on their usual dietary intake and on the execution of the 6MWT, were considered (*n* = 223). Written informed consent was obtained from all participants.

### Dietary information

Information on the subjects’ usual dietary intake was collected by using the Italian version of the validated European Investigation into Cancer and Nutrition (EPIC) FFQ (19). To ensure the quality of participants’ dietary reporting, participants who filled less than 70% of the total number of questions , as well as the subjects with an extremely low caloric intake level and those on top and bottom 0.5% of the distribution of Energy Intake/Basal Metabolism Ratio (EI/BMR), were excluded from the study. Then, daily intake of energy and macronutrients was estimated by means of the NAF software (nutritional analysis of FFQs, National Cancer Institute, Milan, Italy) (20), and the information on nutrients for specific foods consumed in Italy was obtained from the food composition database for epidemiological studies in Italy (21).

### 6MWT

The 6MWT was performed following the American Thoracic Society guidelines (22). Before performing the test, subjects were checked for contraindications. Subjects were asked to walk as fast as they could without running in a 25-m-long hallway, for 6 min. The test results were expressed as the distance walked (6MWD) in metres. Out of 255 controls with available information on their nutritional status, 32 (12.5%) did not perform the 6-min walking test (6-MWT), because of clinical contraindications, including heart attack occurred in the previous 3 months, current drug treatment for epilepsy, a heart rate more than 120 beats per minute and a systolic blood pressure over 180 mmHg or a diastolic blood pressure over 100 mmHg.

### Exposures and potential confounders

Vegetable and animal protein intakes (g/day) were considered as determinants. The following covariates were considered as potential confounders: gender, age, height, weight, smoking habits (never smoker, past smoker, i.e. not smoking in the last month or current smoker), comorbidity, self-reported intensity of physical exercise, total fats and available carbohydrates (i.e. the sum of monosaccharides, disaccharides, dextrins, starch and glycogen expressed in monosaccharides). The distribution of the total energy intake in kJ/day (or in kcal/day) was also calculated. According to the participants’ questionnaire answers, the comorbidity status was defined by the presence of self-reported medical diagnosis of at least one of the following diseases: arterial hypertension, diabetes, cardiovascular comorbidity (at least one of lifetime heart attack, ictus, angina pectoris, arrhythmia, heart or aorta surgery) and cancer. Intensity of exercise performed during a week was classified into three levels and estimated by asking participants how often, and for how many hours weekly they were exercising so as to have a feeling of shortness of breath and to sweat.

### Statistics

Subject characteristics were summarised as percentages or means (SD). A two sample *t*-test on the equality of means was performed to investigate the difference in physical performance (6MWD) between subjects ingesting more or less than 0.8 g protein per kg of body mass-daily, which is the recommended daily allowance (RDA) for proteins (23). Models considering the 6MWD as the dependent variable were fitted using a simple linear regression for the nutrient intakes of interest. Then, multiple linear regression models were fitted to the data, with each nutrient intake as the independent variable, adjusting for a first set of potential confounders (gender, age, height, weight, smoking habits, comorbidities and the self-reported intensity of physical training). Another model was fitted considering, in addition to the first set of confounders, also animal proteins, vegetables proteins, total fats and available carbohydrates. Lastly, a multiple regression model similar to the previous one was fitted taking into account the total proteins (expressed as g/day) instead of vegetable and animal proteins separately. All statistical analyses were performed using the software Stata, version 13.0 (www.stata.com).

## Results

The socio-demographic characteristics of the 223 participants are shown in Table 1. Their mean age at the clinical visit was 45.8 years (SD = 9.6 years; range = 22.8–68.4 years). Most participants were women (57.0%), had a normal mean value of BMI (24.4 kg; range = 16.9–39.8 kg/m), were non-smoker (58.1%) and reported a light intensity of physical training (48.4%) and the absence of comotbidities (75.2%). The mean value of weight and height were 70.6 (range = 41–110) kg and 169.6 (range = 147–192) cm, respectively (Table 1). Their dietary intake is shown in Table 2. The median total energy intake was 7,924.5 kJ/day (= 1,894 kcal/day). The subjects reported daily median intakes of 48.2 g/day for animal proteins (i.e. 0.7 g/kg bw/day) and 24.3 g/day for vegetable proteins (i.e. 0.4 g/kg bw/day). Table 2 reports daily dietary information reported as percentage of total energy intake.

**Table 1:** Socio-demographic characteristics of participants (*N* = 223)^§^

	Participants (*n* = 223)*
Age at the clinical visit, years	45.8; 9.6
Males	43.0 (96)
Females	57.0 (127)
BMI, kg/m^2^	24.4; 4.0
Weight, kg	70.6; 14.8
Height, cm	169.6; 9.4
Non smoker	58.1 (129)
Ex-smoker	28.8 (64)
Current smoker	13.1 (29)
Heavy physical activity	7.6 (17)
Moderate physical activity	44.0 (98)
Light physical activity	48.4 (108)
Presence of comorbidity	24.8 (55)
Absence of comorbidity	75.2 (167)

§ Subject characteristics were summarised as percentages (number of observations) or means (SD) related to qualitative and quantitative variables, respectively.

* There were missing values on smoking habits (one missing value) and comorbidity (one missing value), so the percentages were calculated considering the total number of subjects after excluding the missing data.

**Table 2:** Nutritional intake estimates of participants (*N* = 223)

Nutritional dietary intake	Median	First quartile; third quartile	Range
Vegetable protein – g/day	24.3	18.3; 31.2	8.2–95.7
Animal protein – g/day	48.2	38.1; 63.7	9.5–122.5
Total protein – g/day	73.3	58.5; 93.6	22.7–180.6
Vegetable protein–g/kg/day	0.4	0.3; 0.5	0.1–1.3
Animal protein–g/kg/day	0.7	0.5; 1.0	0.1–2.2
Total proteins–g/kg/day	1.1	0.8; 1.4	0.3–2.7
Available carbohydrates – g/day	235.6	174.4; 308.7	73.1–748.9
Total fats –g/day	74.8	60.6; 95.6	20.6–169.8
Total energy intake – kJ/day [Total energy intake – kcal/day]	7,926.2 [1,894.4]	62,960.8; 103,989.1 [1,504.8; 2,485.4]	2,985.3–19,491.2 [713.5–4,658.5]
Total proteins– energy %	15.5	14.1; 17.3	9.9–26.3
Vegetable protein–energy %	5.1	4.5; 5.8	3.0–8.6
Animal protein–energy %	10.0	8.5; 12.3	3.9–22.7
Available carbohydrates – energy%	49.1	43.2; 54.3	28.4–71.0
Total fats–energy %	35.3	31.5; 38.9	22.5–53.0

**Fig. 1:**
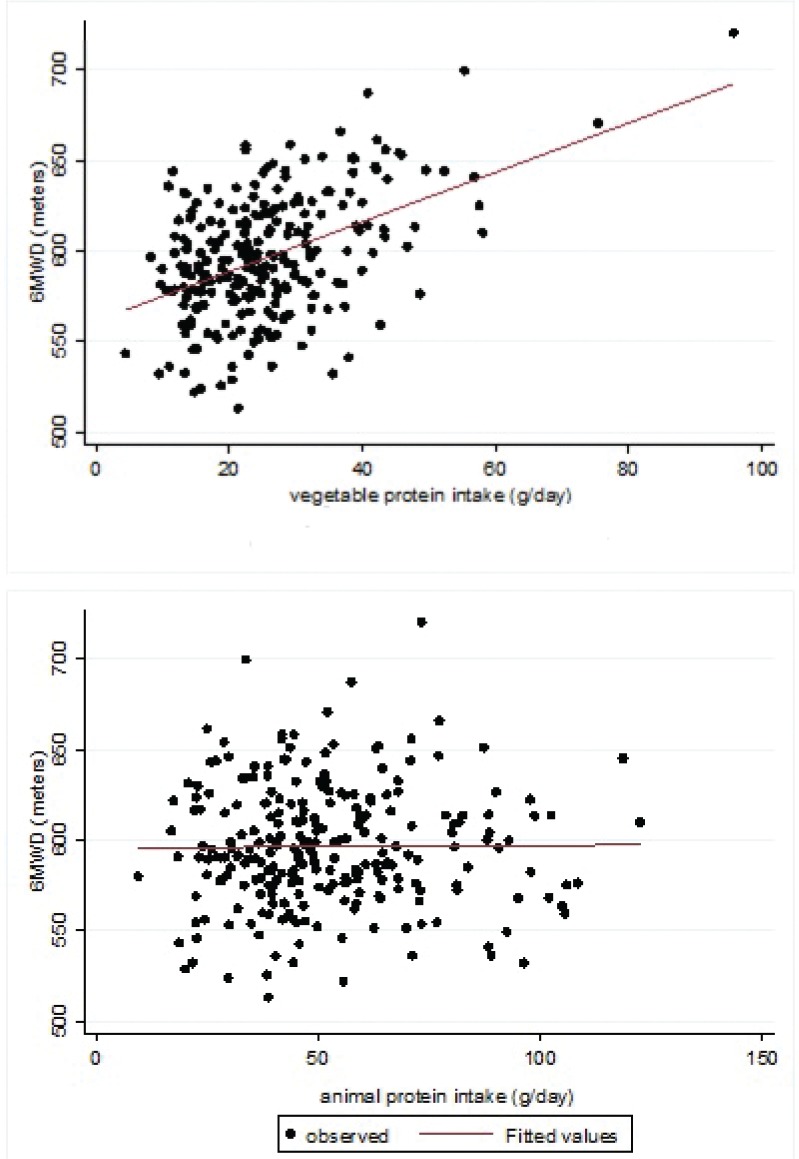
Vegetable protein intake (g/day) or animal protein intake (g/day) and 6MWD (metres). The lines represent the fitted value of the multiple linear regressions where the dependent variable is 6MWD and the independent variable is the vegetable protein intake (upper panel) and the animal protein intake (lower panel). The two models are adjusted for the following covariates: gender, age, height, weight, smoking habits, comorbidities, the intensity of physical training, vegetable protein (g/day), animal protein (g/day), available carbohydrates (g/day) and total fats (g/day).

The majority of the participants (78.9%) reported a protein intake that was more than 0.8 g protein per kg of body mass-daily. The median 6MWD value was 599.4 m (SD = 70.0). There was not a significant difference in the 6MWD between subjects ingesting more or less than 0.8 g protein per kg of body mass-daily (599.8 and 598.1 m, respectively, *P* = 0.89). When considering the simple linear regression, there was a significant increase of 13.2 (95% CI: 5.4; 21.0) m (*P* < 0.001) for 10 g/day increase in the vegetable protein intake (Table 3), and no apparent variation of the 6MWD (−0.3; 95% CI: −4.7; 4.0 m, *P* = 0.881) for 10 g/day increase in the animal protein intake (Table 3). When adjusting for the first set of confounders (gender, age, height, weight, smoking habits, comorbidities and intensity of physical training) and when also adjusting for all the other nutrients (i.e. animal proteins, total fats and available carbohydrates), the positive association between the vegetable protein intake and the 6MWD was confirmed. When considering the latter multivariable regression model, the predicted increase in the 6MWD was 20.0 (95% CI: 0.8; 39.2; *P* = 0.041) m for 10 g/day increase in the vegetable protein intake (Table 3, Fig. 1, upper panel). Total fats (*b* coefficient for 10 g intake increase = −1.4; *P* = 0.69) as well as available carbohydrates (*b* coefficient for 10 g intake increase = −0.9; *P* = 0.44) and animal proteins (*b* coefficient for 10 g intake increase = −1.8; *P* = 0.64) were not associated with the 6MWD (Table 3, last column; Fig. 1, lower panel). Lastly, another multiple regression model was fitted to the data, taking into account the total proteins, instead of vegetable and animal proteins separately, and adjusting for all the above-mentioned confounders, including total fats and available carbohydrates. This model showed that the total protein intake (*b* coefficient for 10 g intake increase = 0.5; 95% CI −6.8; 7.7) was not associated with the 6MWD.

**Table 3:** Simple and multiple linear regression coefficients (with 95% CIS) for regression of 6MWD (M) against nutrient intakes for an increase of 10 g/day

Nutritional dietary intake –10 g/day	*b*-Coefficient (simple linear regression)	Adjusted b-coefficient (*) (multiple linear regression)	Adjusted b-coefficient (**) (multiple linear regression)
Animal protein	−0.3 (−4.7; 4.0)	−1.4 (−5.5; 2.8)	−1.8 (−9.3; 5.7)
Vegetable protein	**13.2 (5.4; 21.0)**	**9.5 (1.7; 17.3)**	**20.0 (0.8; 39.2)**
Total fats	1.5 (−1.9; 4.9)	0.1 (−3.2; 3.3)	−1.4 (−8.4; 5.6)
Available carbohydrates	**1.3 (0.4; 2.3)**	0.8 (−0.1; 1.7)	−0.9 (−3.3; 1.5)

* *b*-Coefficient is adjusted for the following covariates: gender, age, height, weight, smoking habits, comorbidities and the self-reported intensity of physical training;

** *b*-Coefficient is adjusted for the following covariates: gender, age, height, weight, smoking habits, comorbidities, the self-reported intensity of physical training, vegetable protein (g/day), animal protein (g/day), available carbohydrates (g/day) and total fats (g/day). Regression coefficients that are significantly different from 0 are reported in bold.

## Discussion

Our main result was the finding of a direct relationship between the vegetable protein intake and the distance walked in the 6MWT. It is noteworthy to mention that the association persisted after adjusting for several possible confounders. On the contrary, no significant association was found for animal and total protein intakes. Several previous studies (11–14) have been conducted on the association between dietary protein intake and physical performance. Isanejad et al. (13), studying women aged 65–71 years belonging to the Osteoporosis Risk Factor and Prevention Fracture Prevention Study, demonstrated that subjects with a higher protein intake (≥1.2 g/kg) had a significantly better physical function and muscle strength compared with those with moderate and lower intakes. Gregorio et al. (11) demonstrated that upper and lower extremity function was impaired in older women consuming a low protein diet (below the RDA for protein defined as less than 0.8 g protein/kg) compared to those with a higher protein intake, and Radavelli-Bagatini et al. found that dairy protein intake improved physical function in older women (15). Finally, an insufficient consumption of protein has been associated with impairment of physical function and of quality of life in older adults with depression (12). Differently from previous studies (11–13), we found no association between exercise capacity and total protein intake, and we could not demonstrate a different physical performance between subjects ingesting more or less than 0.8 g protein per kg of body mass-daily. Several reasons may justify the contrasting results. The total protein intake of our sample (median value of 1.1 g/kg bw/day) was comparable with the intake of previous studies in Caucasians, ranging from 0.8 to 1.2 g/kg (16, 24). Thus, other factors may account for the discrepant results, as explained in the following. The difference in the results may be attributed to the fact that previous studies were generally carried out on elderly subjects (12–15), who have different skeletal muscle characteristics from those of middle-aged subjects (8). The different methods used to evaluate physical performance, directed to the measurement of strength and balance in previous studies (11–15), and to the evaluation of aerobic capacity in the present one, may also justify the contrasting results. Our data indicate that a higher vegetable protein intake is associated with a better performance at 6MWT. This result was unexpected since animal protein seems to have a higher essential amino acid content and a better protein availability, a characteristic that might ameliorate muscle protein synthesis and anabolism (7, 25). Few studies have been conducted on the separate effect of animal and vegetable protein on physical performance. Houston et al. found that the intake of animal but not vegetable protein was associated with the preservation of lean body mass in a 3-year follow-up study in older adults (16). These results were consistent with those obtained in a cross-sectional study by Lord et al. (26). In addition, the finding that an omnivorous diet increases lean body mass, while lacto-ovo-vegetarian diet results in a loss, though modest, of lean mass in males participating in a programme of resistance training, seems to support an overall better effect of animal proteins on muscle (10). After studying an older group of Chinese community-dwelling people for 4 years, Chan et al. found that a higher protein intake deriving from vegetables, but not from animal source, was associated with the preservation of muscle mass (27). Similarly, in a longitudinal cohort study in Japan, Kojima et al. showed that the age-related decline in muscle strength in women was lower in those who frequently eat soy products or green and yellow vegetables (28). The reasons for our unexpected results are not clear. The association between plant-based proteins and the 6-min walking distance may not be due to the effect of these macro-nutrients on muscle, but it could be related to other components of vegetable foods, such as antioxidants, potentially affecting muscle mass and strength (29). This hypothesis is speculative and warrants further investigation. The present study differs from the previous ones inasmuch the physical performance was evaluated by using the 6MWT, which is influenced not only by muscle strength, but also by cardiovascular and respiratory function (30). While animal proteins seem to improve muscle protein synthesis more than plant proteins (31), a vegetable protein-dietary intake is associated with beneficial cardiovascular effects both in healthy subjects (32) and in patients (33). Particularly, vegetarian dietary practices have been associated with several health benefits, among which are lower risks of dyslipidaemia, hypertension, obesity (34–37) and of chronic diseases in general (32, 38–45). Since many factors related to cardiovascular diseases may negatively affect physical performance (46), it is possible to speculate that the positive association between vegetable protein intake and 6MWD is mediated by a general health benefit rather than through a direct effect on muscle. In other words, a diet rich in vegetable products may be part of a healthy lifestyle and as a consequence of a better physical performance.

The increase in the distance walked by the intake of 10 g/day of vegetable proteins is 20 m in the adjusted model. As expected, this increase is quite small; however, there are no indications on a 6MWD variation describing a meaningful change of performance. Moreover, it is interesting to observe that we found an increase in the distance walked by 10 g/day of vegetable proteins (20 m) comparable to the difference associated with gender (adjusted difference in males with respect to females is 25.3 m). Therefore, we suppose that the increase observed is not negligible.

Our study has some limitations. Dietary assessment by FFQ is subject to the recall bias, so that the measurement error may distort the association between nutrient intakes and outcome measures. On the other hand, the FFQ provided visual aids for the estimation of portions, and this could have been useful to improve the accuracy of the reported information. Moreover, the diet was assessed at a single point in time, a fact that reflects recent rather than long-term exposure; however, there is evidence that adults maintain relatively stable long-term dietary habits (47). We also acknowledge that the study was performed on subjects without respiratory diseases, so the results could not be generalised.

The strengths and originality of this study are that we evaluated a representative sample of middle-aged subjects from the general population and we considered the vegetable and animal protein intake separately, using validated food frequency questionnaires with a careful dietary assessment.

## Conclusions

In conclusion, our population-based study did not show any association between total and animal protein intake and the distance walked in 6 min. On the contrary, a higher protein intake from a vegetable source resulted in a better physical performance. Whether this result is related to the high vegetable protein intake itself or is a consequence of the antioxidant property of plant-based foods or of some beneficial effect associated with a plant-rich dietary pattern, deserves further investigation. However, recommending higher intakes of vegetable protein might be a useful measure for ameliorating physical performance in the general population.
